# Vacancy-Assisted Polarization-Catastrophe Framework for Wake-Up and Ferroelectricity in Hafnium Oxide

**DOI:** 10.1021/acs.nanolett.6c01204

**Published:** 2026-08-27

**Authors:** Agniva Paul, Apu Das, Jonas Müller, Mohit Tewari, Sunanda De, Zhao-Feng Lou, Yannick Raffel, Artur Useinov, Tian-Li Wu, Guilhem Larrieu, Tarun Agarwal, Min-Hung Lee, Sourav De

**Affiliations:** † College of Semiconductor Research, 34881 National Tsing Hua University, Hsinchu 300044, Taiwan; ‡ Institute of Semiconductors and Microsystems, Technische Universität Dresden, 01062 Dresden, Germany; § 54928 LAAS-CNRS, Université de Toulouse, 31031 Toulouse, France; ∥ Department of Electrical Engineering, 242275 Indian Institute of Technology Gandhinagar, Palaj, Gujarat 382055, India; ⊥ Graduate Institute of Electronics Engineering, 33561 National Taiwan University, Taipei 106319, Taiwan; # Fraunhofer IPMS, Center Nanoelectronic Technologies, 01109 Dresden, Germany; & International College of Semiconductor Technology, 34914 National Yang Ming Chiao Tung University, Hsinchu 300, Taiwan; @ Department of Electronics and Electrical Engineering, 34914 National Yang Ming Chiao Tung University, Hsinchu 300, Taiwan

**Keywords:** Hafnium Oxide, Ferroelectricity, Polarization, Polarization Catastrophe,, Vacancy ordering

## Abstract

Ferroelectric wake-up in hafnium oxide remains one of the key unresolved problems in oxide electronics. Here, we combine four-dimensional scanning transmission electron microscopy (4D-STEM), X-ray photoelectron spectroscopy (XPS), first-principles calculations, electrical characterization, and theoretical modeling to investigate wake-up in TaN/Hf_0.5_Zr_0.5_O_2_/TiN capacitors. Although the remanent polarization increases by approximately 2.6-fold after wake-up, the orthorhombic phase fraction changes only slightly from 20.3% to 21.4%, indicating that structural evolution alone cannot explain the electrical activation. XPS and first-principles calculations reveal that the ferroelectric response is highly sensitive to oxygen-vacancy configuration. Motivated by these observations, we develop a vacancy-assisted polarization-catastrophe framework in which vacancy ordering renormalizes the dipolar interaction and drives the transition from a nonpolar single-well to a ferroelectric double-well energy landscape. The model quantitatively explains the observed enhancement of polarization, switching current, capacitance, and hysteresis, establishing vacancy ordering as the governing mechanism of wake-up in hafnium oxide.

## Graphical abstract

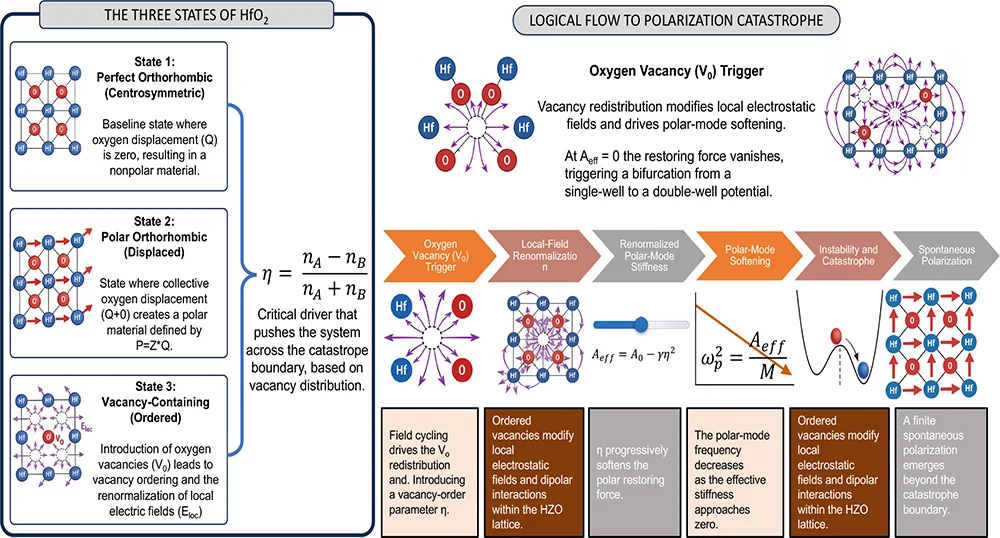

Ferroelectricity is a central topic in condensed-matter physics.^1–4^ Since the earliest theories of dielectric instability, spontaneous polarization has been understood as a balance among electrostatic interactions, lattice restoring forces, and anharmonic stabilization. Lorentz theory showed that the local field acting on a polarizable ion can differ substantially from the applied field, allowing instability when local-field enhancement exceeds the lattice restoring force.^5,6^ This idea later developed into polarization-catastrophe and soft-mode theories of ferroelectric transitions.^7,8^ In conventional perovskites such as BaTiO_3_, PbTiO_3_, and KNbO_3_, ferroelectricity is generally associated with softening of a zone-center transverse optical phonon. Strong oxygen-2p/transition-metal-d hybridization weakens the harmonic restoring force and creates a double-well free-energy landscape. Theories developed by Devonshire, Cochran, Bilz, and others connected lattice dynamics, oxygen polarizability, and local-field effects to spontaneous polarization.^7,9–17^

Ferroelectric HfO_2_ challenges this classical picture. Its stable bulk phase is monoclinic and nonpolar, whereas ferroelectricity requires stabilization of the metastable orthorhombic *Pca*2_1_ phase through film thickness, mechanical confinement, electrode effects, thermal processing, and defect engineering.^16,18–24^ Yet its microscopic origin remains debated. Proposed mechanisms include surface energy, grain-size confinement, interface-induced stress, dopants, and oxygen-vacancy engineering.^16,16,19,20,25^ Oxygen vacancies strongly affect phase formation, wake-up, retention, imprint, and endurance.^14,26–29^ However, whether they merely stabilize the orthorhombic phase or actively govern ferroelectric behavior remains unresolved.^26^ Unlike classical perovskites, hafnia lacks a universally accepted soft-mode mechanism. Although the orthorhombic phase has the symmetry required for polarization, no unified instability model yet explains how the system crosses from a nonpolar state to a polar state while simultaneously incorporating crystal symmetry, local-field interactions, and defects.

Here, we propose a vacancy-assisted polarization-catastrophe framework. The orthorhombic *Pca*2_1_ phase supplies a symmetry-allowed polar coordinate associated with collective oxygen displacement, while vacancy ordering renormalizes local-field interactions and progressively softens the polar mode. Ferroelectricity appears when the vacancy-renormalized restoring force collapses, without requiring a conventional zone-center phonon instability; instead, it arises from cooperative coupling between orthorhombic symmetry and vacancy-induced local-field renormalization. We derive a microscopic Hamiltonian for coupled polarization and vacancy ordering, obtain analytical expressions for the phase boundary, spontaneous polarization, dielectric susceptibility, coercive field, and switching-current response, and compare the predictions with structural and electrical measurements from TaN/HZO/TiN capacitors. The resulting framework provides a physically transparent connection among vacancy ordering, polar-mode softening, and spontaneous polarization in hafnium oxide, thereby reconciling strong electrical activation with modest crystallographic evolution during wake-up and clarifying the active role of vacancies beyond phase stabilization.

Figure 1 summarizes the structural and chemical evolution of TaN/Hf_0.5_Zr_0.5_O_2_ (10 nm)/TiN metal–ferroelectric–metal capacitors during wake-up. X-ray diffraction (XRD), four-dimensional scanning transmission electron microscopy (4D-STEM) with automated crystal orientation mapping (ACOM),^22,30^ and X-ray photoelectron spectroscopy (XPS) were used to correlate crystallographic evolution with oxygen-vacancy chemistry. The XRD pattern in Figure 1b confirms crystallized HZO containing monoclinic and nonmonoclinic phases: the dominant feature near 30°–31° arises from overlapping orthorhombic/tetragonal reflections, whereas the weaker peak near 28° is assigned to the monoclinic phase. These features indicate partial stabilization of the metastable ferroelectric phase, but their overlap prevents reliable XRD quantification of the individual orthorhombic and tetragonal fractions. Therefore, nanoscale phase fractions were determined by 4D-STEM/ACOM. The pristine film contains approximately 21.8% orthorhombic and 78.2% monoclinic phases. After wake-up, the orthorhombic fraction increases only to 22.7%, while the monoclinic fraction decreases to 76.2% and a small tetragonal component emerges. Thus, the orthorhombic fraction increases by only 0.9%, showing that repeated switching produces limited crystallographic evolution. The details of the 4D-STEM are in Section S2.3 in the Supporting Information and also in our previous study.^22^

**Figure 1:**
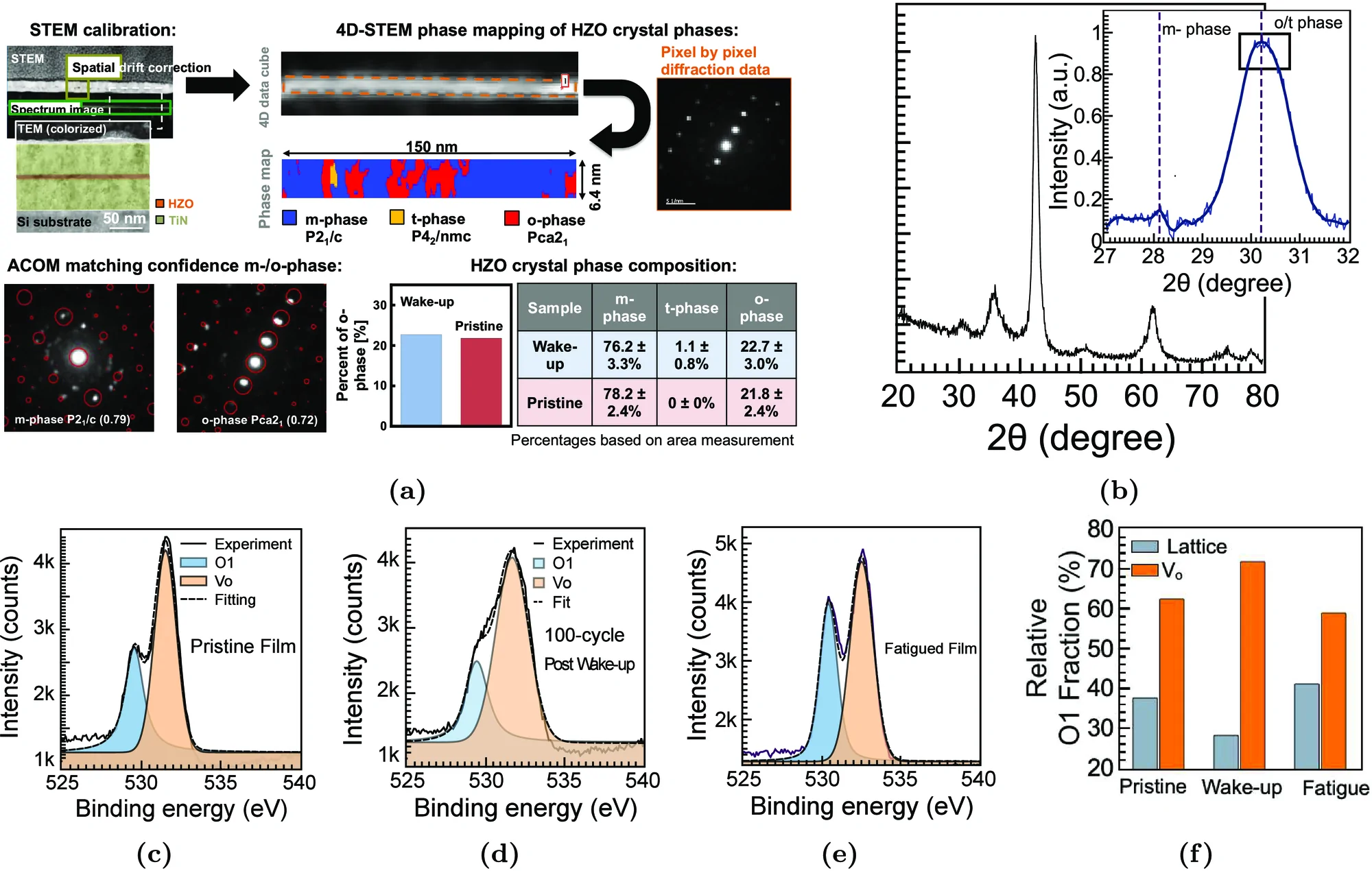
Structural and chemical evolution of HZO thin films during wake-up and fatigue. (a) Cross-sectional STEM/TEM characterization and 4D-STEM phase mapping of the TaN/HZO/TiN capacitor structure. The HZO thickness is ∼9.5 nm. Automated crystal orientation mapping (ACOM) based on 4D-STEM diffraction analysis identifies monoclinic (*m*, *P*21/*c*), tetragonal (*t*, *P*42/*nmc*), and orthorhombic (*o*, *Pca*21) polymorphs within the HZO layer. Averaging the HZO phase composition based on acquired phase maps from pristine and wake-up states reveal only a modest increase in the orthorhombic phase fraction from 21.8% (±2.4%) to 22.7% (±3.0%), while the monoclinic phase decreases from 78.2% (±2.4%) to 76.2% (±3.0%) with the emergence of a minor tetragonal component. Representative HRTEM images and diffraction finger prints used for phase identification are also shown.^22^ (b) XRD pattern of the TiN/HZO/TiN stack with an enlarged view of the 27°–32° region. The dominant reflection near 30°–31° corresponds to the orthorhombic/tetragonal phase region, whereas the weaker feature near 28° is associated with the monoclinic phase. Combined XRD, XPS, and 4D-STEM analyses demonstrate that significant wake-up occurs despite only minor changes in orthorhombic phase fraction, suggesting an important role of defect redistribution in the wake-up mechanism. (c–e) High-resolution O 1s XPS spectra and peak deconvolution of pristine, wake-up (100 cycles), and fatigued HZO films (10^5^ cycles), respectively. The spectra are decomposed into lattice oxygen (O1) and vacancy-related (V_O_) components. Electrical cycling induces a systematic evolution of the O 1s line shape, indicating redistribution of oxygen-related defect states during wake-up and fatigue. Experimental spectra and fitted components are shown by black lines and colored peaks, respectively. (f) Relative fractions of lattice oxygen and vacancy-related O 1s components extracted from integrated peak areas. The evolution of oxygen-related defect states is substantially larger than the corresponding change in orthorhombic phase fraction measured by 4D-STEM.

In contrast, the oxygen-defect chemistry changes markedly. High-resolution O 1s XPS spectra in Figure 1c–e show pronounced evolution of the oxygen-bonding environment from the pristine state through wake-up and fatigue. Deconvolution into lattice-oxygen (O1) and oxygen-vacancy-related (V_O_) components reveals substantial cycling-induced variation in the vacancy-related spectral weight (Figure 1f), indicating redistribution of oxygen-vacancy populations. This chemical evolution is much larger than the change in orthorhombic phase fraction. Hence, electrical cycling produces extensive oxygen-defect redistribution despite only modest crystallographic evolution, indicating that phase transformation alone is unlikely to dominate wake-up and that defect-mediated changes in the local ferroelectric environment are important. Before XPS analysis, the TaN top electrode was completely removed by end point-controlled atomic layer etching, and the O 1s spectra were acquired directly from the exposed HZO surface; probing-depth considerations are given in Section S2.3 in the Supporting Information.

Figure 2 summarizes the corresponding electrical evolution. In the pristine capacitors, the switching-current (*I*_sw_) characteristics and polarization–voltage (*P*–*V*) loops measured with PUND amplitudes of 1–2 V and a fixed pulse width of 500 μs are shown in Figure 2a,b. At low programming voltages, weak switching-current peaks and narrow, partially unsaturated loops indicate incomplete polarization reversal and a limited switchable ferroelectric volume. After wake-up, Figure 2c,d show substantially larger and sharper switching-current peaks, together with wider loops approaching saturation across the programming-voltage range, demonstrating progressive activation of ferroelectric switching.

**Figure 2:**
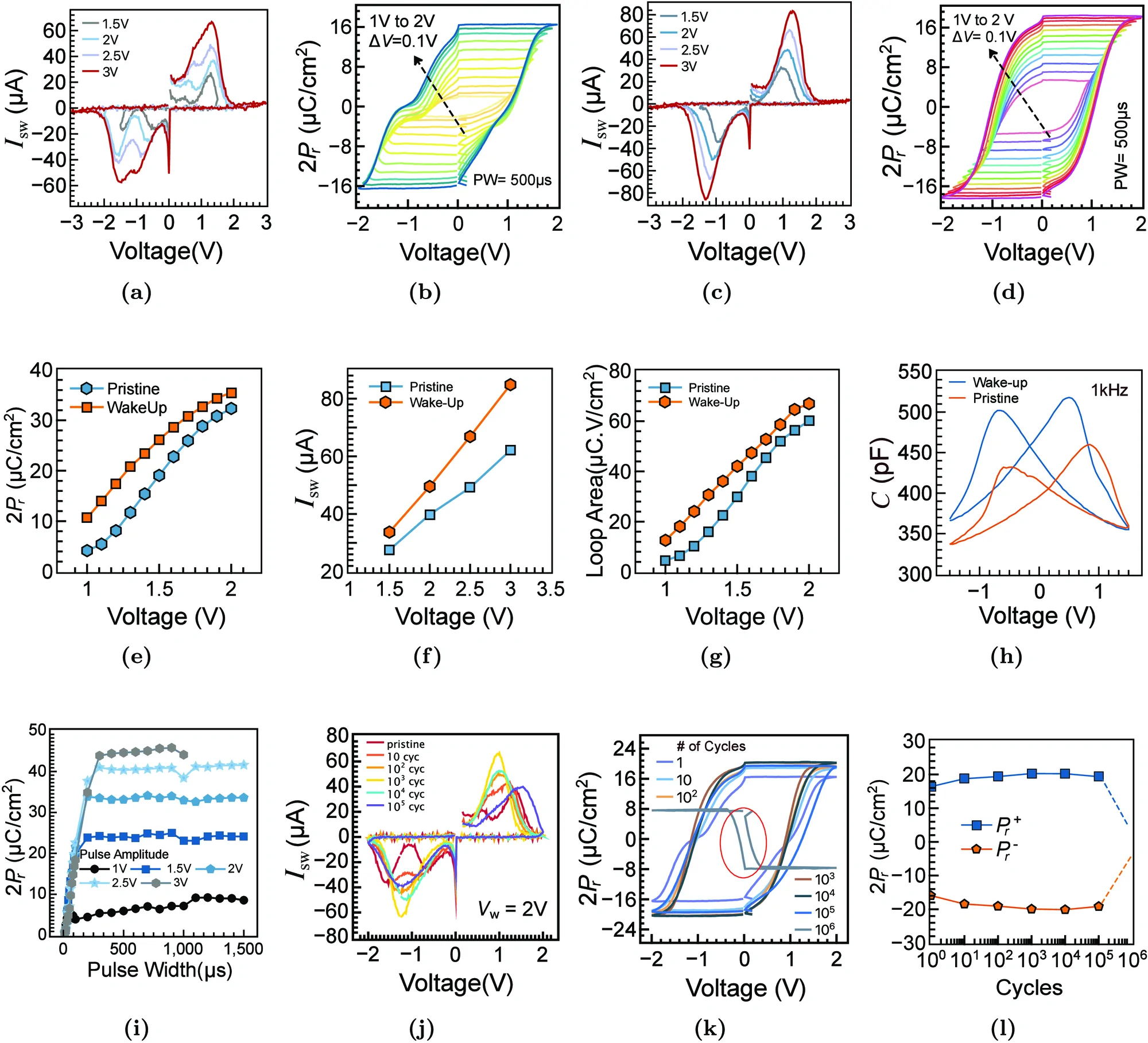
Electrical evolution of HZO capacitors during wake-up, switching, and fatigue. (a,b) Switching-current (*I*_sw_) characteristics and remanent polarization–voltage (*P*_r_–*V*) hysteresis loops of the pristine TaN/HZO/TiN capacitor measured using PUND pulse amplitudes from 1 to 2 V. (c,d) Corresponding *I*_sw_ and *P*_r_–*V* characteristics after wake-up cycling, showing enhanced switching current and enlarged polarization hysteresis. (e) Evolution of remanent polarization (2*P*_r_) as a function of PUND pulse amplitude for pristine and wake-up states. (f) Peak switching current extracted from *I*_sw_ measurements as a function of pulse amplitude. (g) Evolution of the hysteresis-loop area, ∮*P* d*V*, illustrating the progressive activation of ferroelectric switching following wake-up. (h) Comparison of the 1 kHz capacitance–voltage (*C*–*V*) characteristics before and after wake-up, exhibiting the characteristic butterfly response associated with polarization reversal. (i) Dependence of 2*P*_r_ on pulse width for different pulse amplitudes, revealing the switching kinetics and polarization saturation behavior. (j,k) Evolution of the switching-current characteristics and *P*_r_–*V* hysteresis loops during endurance cycling. (l) Positive and negative remanent polarization ( $P_{r}^{+}$ and $P_{r}^{-}$ ) extracted as a function of cycle number. The combined electrical measurements demonstrate that wake-up produces a substantial enhancement of switchable polarization and switching current, despite the relatively minor structural evolution observed in Figure 1.

Figure 2e–g quantify this activation: the remanent polarization (2*P*_r_), peak switching current $\left(I_{sw}^{peak}\right)$ , and hysteresis-loop area increase after wake-up at every investigated programming voltage. In particular, 2*P*_r_ increases by approximately 2.56×, far exceeding the 1.05× increase in orthorhombic phase fraction. Figure 2h provides further evidence through the capacitance–voltage response: wake-up produces a more pronounced butterfly characteristic and higher peak capacitance. Because differential capacitance reflects the local curvature of the ferroelectric free-energy landscape, this increase indicates softening of the effective restoring force acting on the polarization coordinate.

The pulse-width dependence in Figure 2i, measured for programming voltages of 1–3 V, shows that 2*P*_r_ rises rapidly at short pulse widths and then approaches saturation. Both the switching rate and saturation polarization increase with programming voltage, indicating that stronger fields activate a larger fraction of the available switchable polar volume. Figure 2j–l trace the subsequent evolution from wake-up to fatigue. The switching-current peaks in Figure 2j first increase and then decrease after prolonged cycling; the *P*–*V* loops in Figure 2k similarly widen during wake-up and shrink during fatigue. Figure 2l shows that both positive and negative remanent-polarization states increase initially, reach a maximum, and then decline. Collectively, these measurements establish a continuous progression from pristine to wake-up and fatigue. The large electrical enhancement, despite only minor structural change and together with substantial oxygen-defect redistribution, points to defect-mediated modification of the local free-energy landscape rather than crystallographic phase transformation alone. We therefore examine whether progressive vacancy ordering can renormalize the local dipolar interaction, drive a polarization catastrophe, and account for the observed electrical activation.

Figure 3 presents the finite-temperature evolution of oxygen-vacancy configurations and the associated spontaneous polarization of orthorhombic HZO. Figure 3a–d show the initial ordered vacancy configurations used for molecular-dynamics (MD) simulations, whereas Figure 3e–h show the corresponding structures after 75 ps of equilibration at 300 K. Local atomic rearrangements around the vacancies indicate redistribution toward energetically favorable configurations while the overall orthorhombic framework is preserved. Figure 3I compares the normalized polarization before and after equilibration.

**Figure 3:**
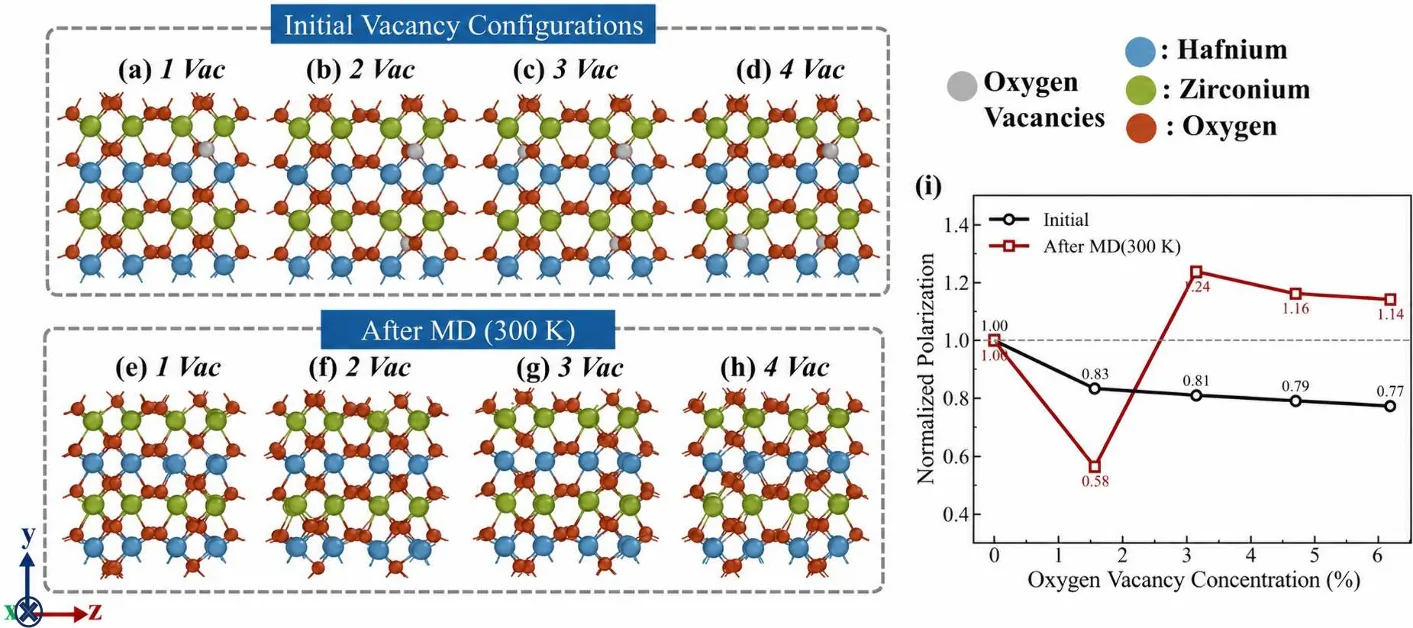
Influence of oxygen-vacancy redistribution on the spontaneous polarization of orthorhombic Hf_0.5_Zr_0.5_O_2_ (HZO). (a–d) Initial ordered oxygen-vacancy configurations containing one, two, three, and four oxygen vacancies, respectively, used as the starting structures for molecular dynamics (MD) simulations. Oxygen vacancies are highlighted by gray spheres. (e–h) Corresponding equilibrium structures obtained after 75 ps of MD equilibration at 300 K using the MACE-medium machine-learned interatomic potential. (i) Normalized spontaneous polarization before and after MD equilibration as a function of oxygen-vacancy concentration. Vacancy redistribution during finite-temperature equilibration significantly modifies the spontaneous polarization, demonstrating that the ferroelectric response depends strongly on the spatial configuration of oxygen vacancies rather than solely on their concentration.

Before MD equilibration, the normalized polarization decreases gradually with increasing oxygen-vacancy concentration, indicating that the imposed initial configurations locally suppress the ferroelectric distortion. After equilibration, vacancy redistribution instead produces a pronounced enhancement: the normalized polarization exceeds that of pristine HZO above approximately 4% vacancy concentration, reaches a maximum near 6%, and then decreases slightly, indicating saturation for the configurations studied. Thus, the ferroelectric response depends on vacancy spatial arrangement as well as concentration. Only one representative configuration was considered at each vacancy concentration; because many crystallographically distinct arrangements are possible, other initial configurations may relax into metastable states with different polarization values. The calculations therefore represent the selected configurations rather than a complete configurational average, but they clearly establish strong coupling between finite-temperature vacancy redistribution and spontaneous polarization. Together with the structural and electrical data, these first-principles results support wake-up as being governed predominantly by changes in local defect configuration rather than extensive crystallographic phase transformation. Microscopic theory of vacancy-assisted polarization catastrophe: The experimental observations together with the first-principles calculations demonstrate that oxygen-vacancy redistribution substantially modifies the ferroelectric response while producing only a minor change in the crystallographic phase fraction. A microscopic description of wake-up must therefore account for two coupled processes: the evolution of the local vacancy configuration during electrical cycling and its influence on the stability of the ferroelectric polar state. To capture this interplay, we formulate a microscopic Hamiltonian in which progressive vacancy ordering continuously renormalizes the dipolar interaction governing the orthorhombic ferroelectric distortion.

The ferroelectric instability of orthorhombic Hf_0.5_Zr_0.5_O_2_ originates from the symmetry-breaking polar distortion of the noncentrosymmetric *Pca*2_1_ crystal structure.^18–20^ Since this distortion is dominated by the collective displacement of oxygen ions relative to the Hf/Zr sublattice, the lattice dynamics can be represented by a coarse-grained local polar-mode coordinate. We therefore introduce a local displacement variable *q*_*i*_, which describes the symmetry-breaking oxygen displacement within unit cell *i*.

The local electric dipole associated with this polar distortion is

1 $$p_{i}=Z^{*}q_{i}$$

where Z* denotes the Born effective charge of the polar mode.

Summing over all unit cells gives the macroscopic polarization,

2 $$P=\frac{1}{\Omega }\underset{i}{\sum }p_{i}=\frac{Z^{*}}{\Omega }\underset{i}{\sum }q_{i}$$

where Ω is the crystal volume. Introducing the uniform collective displacement *Q* = ⟨*q_i_* ⟩ yields

3 $$P=\frac{Z^{*}}{\Omega }Q$$

thereby establishing *Q* as the macroscopic order parameter of the ferroelectric state.

The first-principles calculations together with the XPS measurements indicate that the dominant microscopic evolution during wake-up is not the creation of oxygen vacancies but their redistribution within the orthorhombic lattice. Consequently, the relevant microscopic variable is not the total vacancy concentration but the degree of vacancy ordering, which governs the local electrostatic environment experienced by the polar mode. To describe this evolution, we introduce a local occupation variable

4 $$n_{i}=\{\begin{array}{ll} 1 & \mathrm{oxygen vacancy at site}\;i\\ 0 & \mathrm{otherwise} \end{array}$$

and define the corresponding coarse-grained vacancy-order parameter

5 $$\eta =\frac{n_{A}-n_{B}}{n_{A}+n_{B}}$$

where *n*_A_ and *n*_B_ denote the populations of oxygen vacancies occupying two symmetry-related oxygen sublattices. Unlike the overall vacancy concentration, which remains approximately conserved during electrical cycling, η characterizes the degree of vacancy ordering. A completely random vacancy distribution corresponds to η = 0, whereas increasing preferential occupation of one symmetry-equivalent sublattice produces progressively larger values of η. Within the present framework, η therefore serves as the microscopic control parameter governing the evolution of the ferroelectric state during wake-up.

Having identified the relevant microscopic degrees of freedom, we now formulate the Hamiltonian governing their coupled evolution. The coupled polar-vacancy system is described by

6 $$H=H_{loc}+H_{dd}$$

where *H*_loc_ describes the local lattice potential and *H*_dd_ represents the long-range dipolar interaction responsible for stabilizing the ferroelectric state.

The local lattice potential represents the intrinsic restoring force acting on the polar distortion and is described using the Landau–Devonshire expansion,

7 $$H_{loc}=\underset{i}{\sum }\left[\frac{1}{2}a_{0}q_{i}^{2}+\frac{1}{4}bq_{i}^{4}+\frac{1}{6}cq_{i}^{6}\right]$$

where *a*_0_ is the harmonic restoring coefficient, while *b* and *c* describe the anharmonic stabilization of the ferroelectric phase.

The second contribution originates from the long-range electrostatic interaction between neighboring dipoles,

8 $$H_{dd}=-\frac{1}{2}\underset{i\neq j}{\sum }J_{ij}p_{i}p_{j}$$

where *J*_*ij*_ denotes the effective dipolar coupling between unit cells *i* and *j*. Substituting eq 1 gives

9 $$H_{dd}=-\frac{1}{2}\underset{i\neq j}{\sum }J_{ij}{\left(Z^{*}\right)}^{2}q_{i}q_{j}$$

Because the dipolar interaction is long-ranged, it is convenient to express the interaction in reciprocal space,

10 $$H_{dd}=-\frac{1}{2}\underset{k}{\sum }J\left(k\right){\left(Z^{*}\right)}^{2}q_{k}q_{-k}$$

The ferroelectric instability is governed by the uniform (**k** = 0) soft mode, for which the effective harmonic stiffness becomes

11 $$A_{FE}=a_{0}-J\left(0\right){\left(Z^{*}\right)}^{2}$$

The quantity *A*_FE_ therefore represents the effective stiffness of the ferroelectric polar mode in the absence of vacancy ordering.

The central hypothesis of the present work is that oxygen-vacancy ordering modifies the local dipolar interaction rather than directly generating spontaneous polarization. As electrical cycling progressively redistributes oxygen vacancies, the local electrostatic environment evolves continuously, leading to a renormalization of the dipolar coupling responsible for stabilizing the ferroelectric state. To the lowest order, this effect can be expressed as

12 $$J\left(0,\eta \right)=J_{0}+J_{2}\eta^{2}+O\left(\eta^{4}\right)$$

Substituting eq 12 into the expression for the harmonic stiffness gives

13 $$A_{e\mathrm{ff}}=a_{0}-{\left(Z^{*}\right)}^{2}J\left(0,\eta \right)$$

Introducing the reference stiffness

14 $$A_{0}=a_{0}-{\left(Z^{*}\right)}^{2}J_{0}$$

and defining the vacancy-induced renormalization coefficient,

15 $$\gamma ={\left(Z^{*}\right)}^{2}J_{2}$$

the effective stiffness becomes

16 $$A_{e\mathrm{ff}}=A_{0}-\gamma \eta^{2}$$

which constitutes the central result of the present theory. Rather than directly generating spontaneous polarization, oxygen-vacancy ordering progressively reduces the restoring force acting on the ferroelectric polar mode through the renormalization of the dipolar interaction. As the degree of vacancy ordering increases, the effective stiffness decreases continuously, driving the system toward a local instability. The corresponding evolution of the dipolar interaction, normalized polar stiffness, and soft-mode frequency is illustrated in Figure 4a.

**Figure 4:**
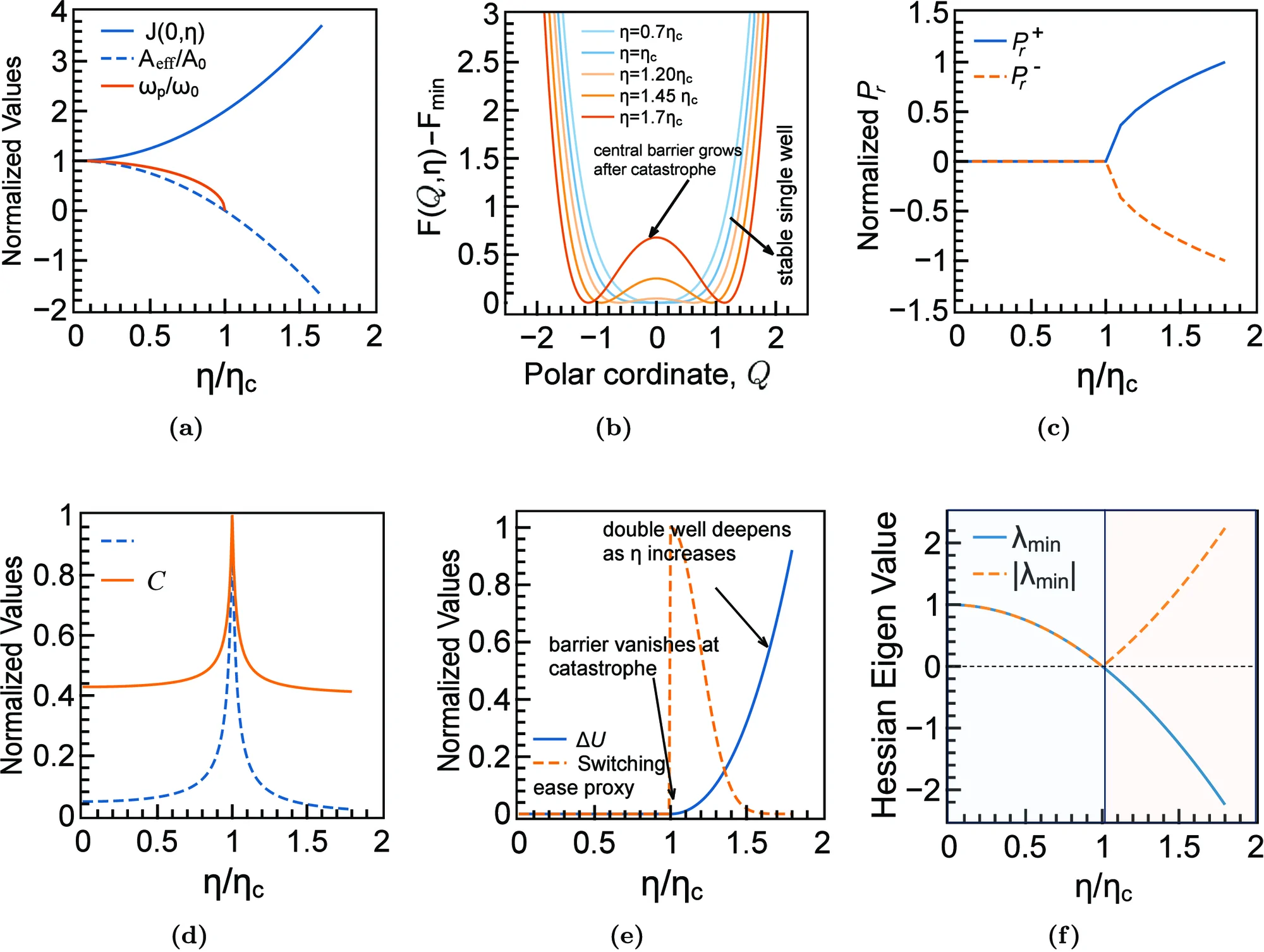
Vacancy-assisted polarization-catastrophe model for ferroelectricity in HZO. (a) Vacancy-induced local-field enhancement *J*(0,η) and renormalization of the polar stiffness *A*_eff_ = *A*_0_ – γη^2^. The polarization catastrophe occurs when *A*_eff_ → 0, corresponding to the softening of the polar mode frequency ω_p_. (b) Evolution of the free-energy landscape with increasing vacancy-order parameter η. A stable single-well potential transforms into a double-well ferroelectric state after the catastrophe condition is reached, accompanied by the emergence of a finite polarization minimum. (c) Development of spontaneous or remanent polarization *P*_r_ following the catastrophe transition. Above the critical vacancy-order parameter η_c_, finite positive and negative polarization states appear continuously from the nonpolar state. (d) Enhancement of dielectric susceptibility and capacitance near the catastrophe boundary. The collapse of the restoring force produces a strong increase in the polar response, resulting in a capacitance maximum close to η = η_c_. (e) Evolution of the switching barrier Δ*U* and the corresponding switching ease. The barrier vanishes at the instability point and subsequently increases as the ferroelectric double well deepens, while switching becomes progressively more difficult away from the critical region. (f) Stability phase diagram expressed through the smallest Hessian eigenvalue λ_min_. The condition λ_min_ = 0 defines the polarization-catastrophe boundary separating the stable nonpolar state (*A*_eff_ > 0) from the ferroelectric state (*A*_eff_ < 0).

Replacing the microscopic displacement field by the collective order parameter *Q* yields the coarse-grained free energy

17 $$F\left(Q,\eta \right)=\frac{1}{2}\left(A_{0}-\gamma \eta^{2}\right)Q^{2}+\frac{1}{4}BQ^{4}+\frac{1}{6}CQ^{6}-EZ^{*}Q$$

For small η, the quadratic coefficient remains positive and the free-energy landscape has a single minimum at *Q* = 0. As vacancy ordering increases, the coefficient *A*_0_ – γη^2^ decreases continuously. The instability occurs when the curvature at the origin vanishes,

18 $$A_{e\mathrm{ff}}=A_{0}-\gamma \eta^{2}=0$$

The corresponding critical vacancy-order parameter is therefore

19 $$\eta_{c}=\sqrt{\frac{A_{0}}{\gamma }}$$

For η > η_c_, the origin becomes unstable and the free-energy landscape evolves from a single-well potential into a double-well ferroelectric potential. This transition defines the vacancy-assisted polarization catastrophe. The evolution of the free-energy landscape with increasing η is shown in Figure 4b.

The equilibrium polar state is obtained by minimizing the free energy with respect to the collective coordinate,

20 $$\frac{\partial F}{\partial Q}=0$$

For the nonzero polar solution, this condition gives

21 $$CQ^{4}+BQ^{2}+\left(A_{0}-\gamma \eta^{2}\right)=0$$

The corresponding sign-resolved spontaneous polarization is therefore

22 $$P_{r}^{\pm }=\pm \frac{Z^{*}}{\Omega }{\left[\frac{-B+\sqrt{B^{2}-4C\left(A_{0}-\gamma \eta^{2}\right)}}{2C}\right]}^{1/2}$$

This expression shows that finite spontaneous polarization emerges only after the vacancy-ordering parameter exceeds the critical value η_c_. Above this boundary, positive and negative polarization states bifurcate continuously from the nonpolar state, as illustrated in Figure 4c. The evolution is continuous because vacancy ordering progressively renormalizes the effective polar stiffness rather than inducing an abrupt structural phase transformation.

Beyond the emergence of spontaneous polarization, the model also predicts a pronounced enhancement of the dielectric response. Since the dielectric susceptibility is determined by the curvature of the free-energy landscape,

23 $$\chi^{-1}=\frac{\partial^{2}F}{\partial Q^{2}}$$

the progressive reduction of the effective stiffness produces

24 $$\chi \propto \frac{1}{A_{e\mathrm{ff}}}$$

Consequently, the capacitance is expected to increase as the system approaches the instability boundary. The corresponding evolution of the normalized dielectric susceptibility and capacitance is illustrated in Figure 4d, where both quantities exhibit the characteristic enhancement associated with polar-mode softening.

The reduction of the free-energy curvature simultaneously lowers the energy barrier separating the two polarization states. As the vacancy-order parameter approaches the critical value, the switching barrier collapses continuously, facilitating polarization reversal under an external electric field. This behavior naturally explains the experimentally observed increase in switching current and remanent polarization during wake-up. The calculated evolution of the normalized switching barrier is shown in Figure 4e, illustrating the progressive reduction of the activation energy as the polarization catastrophe is approached.

The overall stability of the coupled polar-vacancy system is summarized in Figure 4f. The boundary separating the paraelectric and ferroelectric states is determined by the condition

25 $$A_{e\mathrm{ff}}=0$$

which corresponds to the complete softening of the ferroelectric polar mode. Below this boundary the free-energy landscape contains a single stable minimum at *Q* = 0, whereas above it the system evolves into a stable double-well potential supporting spontaneous/remanent polarization. The stability diagram therefore defines the vacancy-assisted polarization catastrophe and provides the theoretical phase boundary governing wake-up in orthorhombic Hf_0.5_Zr_0.5_O_2_.

The theoretical framework predicts that vacancy redistribution acts as a nonlinear control parameter for the ferroelectric response. In this picture, the orthorhombic phase provides the symmetry-allowed polar coordinate, while oxygen-vacancy ordering renormalizes the effective polar stiffness according to

26 $$A_{e\mathrm{ff}}\left(\eta \right)=A_{0}-\gamma \eta^{2}$$

As the system approaches the catastrophe condition *A*_eff_ → 0, small changes in vacancy ordering can produce disproportionately large changes in polarization, switching current, capacitance, and hysteresis. Figure 5 compares these predictions with the experimental observations.

**Figure 5:**
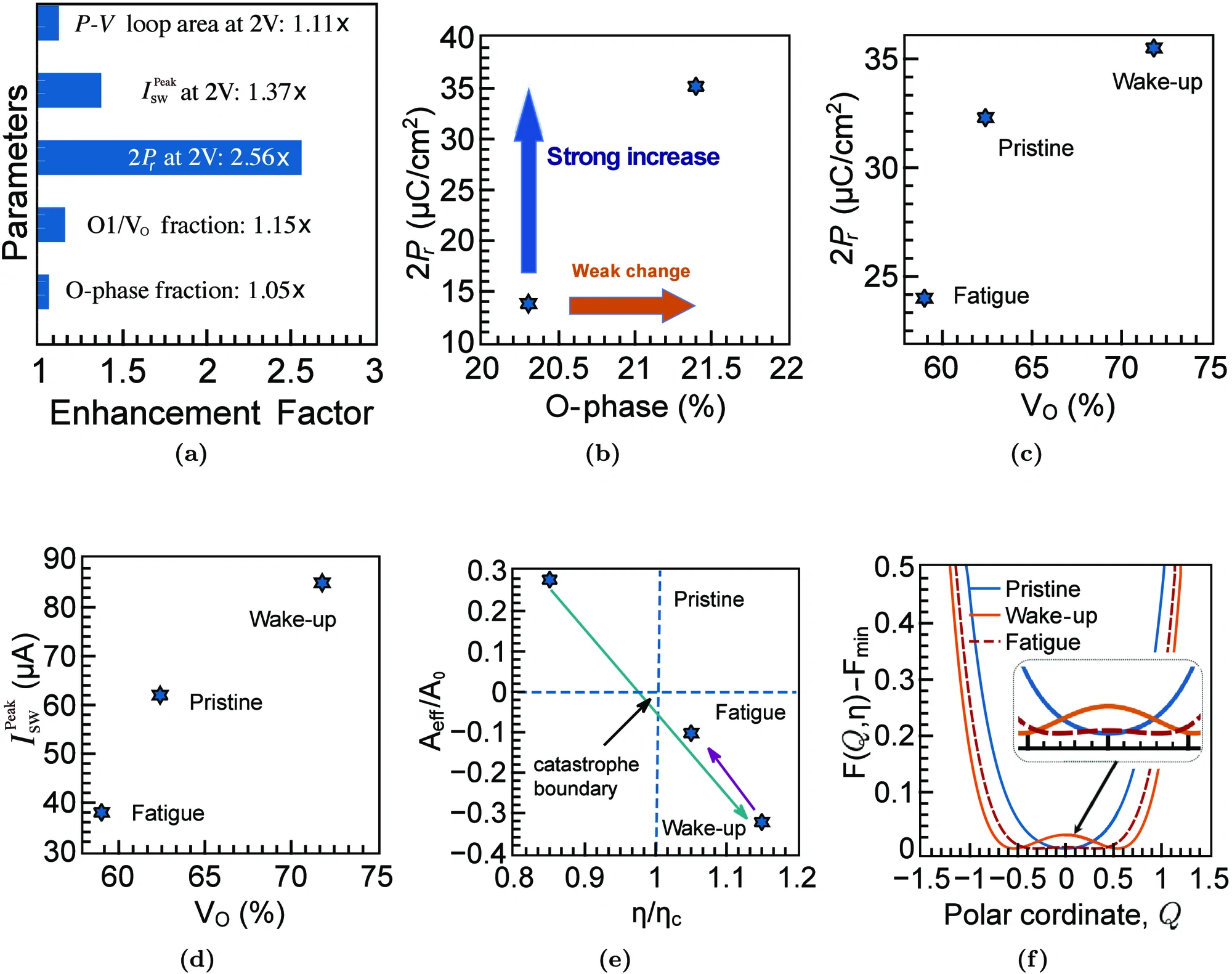
Experimental validation of the vacancy-assisted polarization-catastrophe framework. (a) Relative enhancement factors extracted after wake-up cycling, comparing structural, chemical, and electrical observables. The enhancement of 2*P*_r_ and $I_{\mathrm{sw}}^{\mathrm{Peak}}$ substantially exceeds the corresponding changes in orthorhombic phase fraction and oxygen-vacancy concentration, indicating a highly nonlinear amplification of the ferroelectric response. (b) Correlation between remanent polarization and orthorhombic phase fraction. Despite only a minor increase in the orthorhombic phase content, a pronounced enhancement of switchable polarization is observed, suggesting that wake-up cannot be explained solely by phase transformation. (c) Relationship between remanent polarization and oxygen-vacancy-related O 1s fraction extracted from XPS analysis. The polarization increase closely follows the evolution of vacancy-related defect states. (d) Peak switching current as a function of oxygen-vacancy fraction. The strong increase in $I_{\mathrm{sw}}^{\mathrm{Peak}}$ with vacancy concentration is consistent with polar-mode softening and reduced switching barriers. (e) Conceptual trajectory of pristine, wake-up, and fatigue states in the vacancy-ordering phase space. Increasing vacancy ordering drives the system across the catastrophe boundary (*A*_eff_ = 0), where the renormalized polar stiffness changes sign and a ferroelectric instability emerges. Fatigue shifts the system away from the optimum wake-up condition through defect redistribution and polarization pinning. (f) Corresponding evolution of the free-energy landscape. Wake-up drives the system toward the instability region characterized by a softened double-well potential and enhanced polar susceptibility, whereas fatigue partially restores the switching barrier despite the persistence of oxygen-vacancy defects. Together, these results demonstrate that vacancy redistribution acts as a nonlinear control parameter linking the weak structural evolution observed in Figure 1 to the strong electrical enhancement observed in Figure 2.

Figure 5a summarizes the relative enhancement factors extracted after wake-up cycling. The orthorhombic phase fraction increases only modestly, from 20.3% to 21.4%, corresponding to an enhancement factor of approximately 1.05×. In contrast, the remanent polarization, switching-current peak, and hysteresis-loop area exhibit substantially larger increases. If wake-up were governed primarily by conversion into the orthorhombic phase, the electrical response would be expected to scale approximately with the increase in orthorhombic volume fraction.^14,26^ The measured amplification therefore indicates that an additional microscopic variable beyond phase fraction controls the electrical activation.

This conclusion is reinforced by the weak correlation between orthorhombic phase fraction and remanent polarization shown in Figure 5b. Although the orthorhombic fraction changes only slightly during wake-up, 2*P*_r_ increases much more strongly. By contrast, the remanent polarization follows the evolution of the vacancy-related O 1*s* component extracted from XPS more closely (Figure 5c). Within the present framework, this XPS-derived component is not treated as a direct measurement of η, but rather as an experimental proxy for oxygen-defect redistribution. Its correlation with 2*P*_r_ supports the interpretation that vacancy redistribution modifies the local electrostatic environment and renormalizes the stiffness of the polar coordinate.

The same trend is observed in the switching dynamics. Figure 5d shows that the peak switching current increases with the vacancy-related O 1*s* fraction. Since the switching current is related to the time evolution of the polarization,

27 $$I_{sw}\left(t\right)=A_{dev}\;\frac{dP}{dt}=A_{dev}\frac{Z^{*}}{\Omega }\;\frac{dQ}{dt}$$

where *A*_dev_ is the capacitor area, the increase in *I*_sw_ reflects enhanced motion of the polar coordinate during field-driven reversal. As *A*_eff_ decreases, the free-energy landscape becomes flatter near the instability boundary, reducing the switching barrier and increasing the switching-current response.

Figure 5e maps the pristine, wake-up, and fatigue states onto the vacancy-ordering phase space. In the pristine state, the effective polar stiffness remains positive and the system lies on the stable side of the catastrophe boundary. Electrical cycling redistributes vacancies and drives the system toward

28 $$A_{e\mathrm{ff}}=A_{0}-\gamma \eta^{2}=0$$

The wake-up state corresponds to the regime in which the polar stiffness is strongly reduced, enabling a large increase in switchable polarization. Fatigue is interpreted as a subsequent departure from the optimum vacancy configuration through defect clustering, trapping, interfacial degradation, or polarization pinning. Wake-up and fatigue therefore represent successive stages of defect-mediated evolution within the same local free-energy landscape.

The corresponding free-energy interpretation is shown in Figure 5f. In the pristine state, the energy landscape remains relatively stiff and the accessible polarization response is limited. During wake-up, vacancy redistribution reduces the polar stiffness and drives the system toward a softened double-well landscape, enabling enhanced remanent polarization, larger switching current, and increased capacitance. Upon fatigue, the ferroelectric response decreases despite the continued presence of oxygen-related defects, indicating that not all vacancy configurations promote ferroelectricity equally. Ordered or field-aligned vacancy redistribution can enhance local-field renormalization and polar softening, whereas excessive disorder, clustering, or defect pinning suppresses reversible switching.

Together, these correlations establish the experimental basis for the vacancy-assisted polarization-catastrophe framework. The orthorhombic phase supplies the symmetry-allowed polar coordinate, but the magnitude of wake-up is governed primarily by vacancy-induced renormalization of the local ferroelectric energy landscape. The observed enhancement of 2*P*_r_, *I*_sw_, loop area, and capacitance therefore follows naturally from the approach toward the catastrophe condition *A*_eff_ → 0.

In this work, X-ray diffraction, 4D-STEM, X-ray photoelectron spectroscopy, first-principles calculations, comprehensive electrical characterization, and microscopic theory were combined to investigate ferroelectric wake-up in TaN/Hf_0.5_Zr_0.5_O_2_/TiN capacitors. During wake-up, the orthorhombic phase fraction increases only marginally from 20.3% to 21.4%, whereas the remanent polarization, switching current, hysteresis-loop area, and dielectric response increase substantially. XPS simultaneously reveals pronounced evolution of oxygen-vacancy-related defect states during wake-up and fatigue, showing that defect redistribution is considerably more dynamic than the accompanying crystallographic transformation. Structural phase evolution alone therefore cannot explain the magnitude of the electrical activation. To resolve this discrepancy, we developed the vacancy-assisted polarization-catastrophe framework summarized schematically in Figure 5. In this model, vacancy ordering acts as a hidden control parameter that renormalizes the dipolar interaction and stabilizes the orthorhombic ferroelectric state. Electrical cycling redistributes oxygen vacancies rather than directly generating spontaneous polarization, and the resulting change in the local electrostatic environment progressively reduces the effective polar stiffness. As this stiffness approaches zero, the free-energy landscape evolves from a single-well to a double-well potential, producing enhanced spontaneous polarization, switching current, dielectric susceptibility, and reduced switching barriers, in agreement with the experimental and first-principles results. Ferroelectric wake-up is therefore attributed primarily to vacancy-mediated renormalization of an existing polar framework rather than large-scale orthorhombic phase conversion. Oxygen vacancies not only assist phase stabilization but also continuously tune the local free-energy landscape, explaining how small structural changes can produce disproportionately large electrical responses.

The fatigue response, however, should not be attributed exclusively to vacancy disordering or clustering. Although no discernible additional interfacial layer or structurally distinct dead layer was observed within the examined 4D-STEM regions after cycling, the present measurements cannot exclude an ultrathin electrically inactive interface, subtle interfacial chemical modification, or charge-trapping effects below the sensitivity of the structural analysis. Within the vacancy-assisted framework, prolonged cycling may move the defect configuration away from the optimum field-conditioned state established during wake-up, thereby increasing polarization pinning and suppressing reversible switching. Vacancy redistribution or clustering is therefore considered a plausible contributor acting together with charge trapping, domain pinning, and interfacial degradation, rather than an exclusively established fatigue mechanism. More broadly, the proposed framework unifies crystallographic evolution, oxygen-vacancy dynamics, first-principles calculations, and macroscopic electrical behavior within a single microscopic description. It further suggests that controlling vacancy ordering, rather than only vacancy concentration, may provide a general strategy for engineering polarization switching, dielectric response, and functional stability in metastable oxide ferroelectrics and related polar materials.

## Supplementary Material



## Data Availability

The data and resources that support the findings of this study are available from the corresponding authors upon reasonable request.

## Notes

Complete derivation of the vacancy-assisted polarization-catastrophe framework; collective polar-coordinate and vacancy-order-parameter definitions; microscopic Hamiltonian and vacancy-induced renormalization of polar-mode stiffness; free-energy construction, equilibrium solutions, stability analysis, catastrophe boundary, soft-mode frequency, dielectric susceptibility, switching barrier, and switching-current relations; numerical implementation, parameter normalization, computational workflow, and model parameters; device fabrication and electrical characterization procedures; wake-up, fatigue, capacitance–voltage, and switching-kinetics measurements; X-ray diffraction, TEM, and 4D-STEM structural and phase-composition analyses; HAXPES characterization of oxygen-related chemical states; molecular-dynamics and density-functional-theory calculations of vacancy-dependent ferroelectric polarization (PDF)
