# Supplementary material for: Vacancy-Assisted Polarization-Catastrophe Framework for Wake-Up and Ferroelectricity in Hafnium Oxide
Source: Nano Lett. 2026 Aug 27;26(35):11645–54. doi: 10.1021/acs.nanolett.6c01204 (PMC13564962; doi:10.1021/acs.nanolett.6c01204)
Supplement: Supplementary file 1 [file nl6c01204_si_001.pdf]

Supporting Information: Vacancy-Assisted Polarization-Catastrophe Framework for Wake-Up and Ferroelectricity in Hafnium Oxide

Agniva Paul[1,**], Apu Das[1,**], Jonas Müller[3], Mohit Tewari[4], Sunanda De[2], Zhao-Feng Lou[5], Yannick Raffel[6], Artur Useinov[7,*], Tian-Li Wu[8], Guilhem Larrieu[3], Tarun Agarwal[4], Min-Hung Lee[5], and Sourav De[1,*]

[1] *College of Semiconductor Research, National Tsing Hua University, Hsinchu 300044, Taiwan*
[2] *Institute of Semiconductors and Microsystems, Technische Universität Dresden, 01062 Dresden, Germany*
[3] *LAAS-CNRS, Université de Toulouse, 31031 Toulouse, France*
[4] *Department of Electrical Engineering, Indian Institute of Technology Gandhinagar, Palaj, Gujarat 382055, India*
[5] *Graduate Institute of Electronics Engineering, National Taiwan University, Taipei 106319, Taiwan*
[6] *Fraunhofer IPMS, Center Nanoelectronic Technologies, 01109 Dresden, Germany*
[7] *International College of Semiconductor Technology, National Yang Ming Chiao Tung University, Hsinchu 300, Taiwan*
[8] *Department of Electronics and Electrical Engineering, National Yang Ming Chiao Tung University, Hsinchu 300, Taiwan*
[*] Corresponding authors: *sourav.de@mx.nthu.edu.tw,artu@nycu.edu.tw*
[**] Equal Contribution

Supplementary Note 1. Complete Derivation of the Vacancy-Assisted Polarization-Catastrophe Framework

The theoretical framework presented in the main manuscript describes the evolution of ferroelectric polarization through vacancy-induced renormalization of the polar-mode stiffness. To maintain the concise presentation of the main text, several intermediate mathematical steps and assumptions were omitted. This Supplementary Note provides the complete derivation of the governing equations together with the physical assumptions employed throughout the model.

The present theory is developed for the orthorhombic $Pca2_1$ phase of hafnium oxide, which is the only experimentally established ferroelectric phase. The model assumes that electrical cycling primarily redistributes pre-existing oxygen vacancies rather than inducing a large-scale structural transformation between crystallographic phases. Consequently, the wake-up phenomenon is described as a continuous modification of the free-energy landscape within an already existing orthorhombic lattice.

Throughout this derivation, the microscopic variables are first introduced at the unit-cell level and subsequently coarse-grained into a macroscopic order parameter describing the collective ferroelectric state.

S1.1 Collective Polar Coordinate

The elementary microscopic degree of freedom is the relative displacement of oxygen ions with respect to the surrounding hafnium framework inside the i-th unit cell. This displacement is represented by the local polar coordinate

$$q_i. \tag{S1}$$

The associated local dipole moment is

$$p_i = Z^* q_i, \tag{S2}$$

where Z^* denotes the Born effective charge associated with the ferroelectric soft mode.
The macroscopic polarization is defined as the dipole moment per unit volume,

$$P = \frac{1}{\Omega} \sum_{i=1}^{N} p_i, \tag{S3}$$

where N is the total number of unit cells and Ω is the total crystal volume.
Substituting Eq. (S2) into Eq. (S3) gives

$$P = \frac{Z^*}{\Omega} \sum_{i=1}^{N} q_i. \tag{S4}$$

To describe the collective polarization state, we introduce the average polar displacement

$$Q = \langle q_i \rangle = \frac{1}{N} \sum_{i=1}^{N} q_i. \tag{S5}$$

Therefore,

$$\sum_{i=1}^{N} q_i = NQ. \tag{S6}$$

Substituting Eq. (S6) into Eq. (S4),

$$P = \frac{NZ^*}{\Omega} Q. \tag{S7}$$

The total crystal volume may be written as

$$\Omega = N\Omega_0, \tag{S8}$$

where Ω_0 denotes the primitive unit-cell volume.
Substituting Eq. (S8) into Eq. (S7) gives

$$P = \frac{Z^*}{\Omega_0} Q. \tag{S9}$$

For notational simplicity, the unit-cell volume Ω_0 is denoted as Ω throughout the remainder of the manuscript, resulting in

$$P = \frac{Z^*}{\Omega} Q. \tag{S10}$$

Equation (S10) establishes the direct relationship between the microscopic collective displacement Q and the experimentally measurable macroscopic polarization.

S1.2 Vacancy Ordering Parameter

Electrical cycling redistributes oxygen vacancies within the orthorhombic lattice. Rather than considering only the total vacancy concentration, the present model introduces a quantity describing the spatial asymmetry of vacancy occupation.

The vacancy occupation of the i-th lattice site is represented by

$$n_i = \begin{cases} 1, & \text{vacancy present,} \\ 0, & \text{vacancy absent,} \end{cases} \tag{S11}$$

where n_i is a binary occupation variable.

Two symmetry-related oxygen environments are denoted as A and B. The corresponding coarse-grained vacancy-order parameter is defined as

$$\eta = \frac{n_A - n_B}{n_A + n_B}. \tag{S12}$$

Here,

- $\eta = 0$ corresponds to a statistically symmetric vacancy distribution,
- $\eta > 0$ represents increasing vacancy ordering.

It should be emphasized that η is an order parameter describing vacancy redistribution rather than the total oxygen-vacancy concentration measured experimentally. Experimental techniques such as X-ray photoelectron spectroscopy (XPS) provide information on vacancy concentration but do not directly determine the microscopic ordering parameter introduced here.

The underlying physical assumption of the present model is that the wake-up process predominantly originates from vacancy redistribution occurring during repeated electrical cycling, whereas the total vacancy concentration changes only weakly.

S1.3 Summary of Variables

Table S1 summarizes the principal variables used throughout the theoretical derivation.

Table 1: Definitions of the variables employed in the theoretical framework.

Symbol	Physical meaning	Unit
q_i	Local oxygen displacement	Å
Q	Collective polar coordinate	Å
p_i	Local dipole moment	C·m
P	Macroscopic polarization	$\mathrm{C\,m^{-2}}$
Z^*	Born effective charge	e
Ω	Unit-cell volume	$\mathrm{m^3}$
n_i	Vacancy occupation variable	Dimensionless
η	Vacancy-order parameter	Dimensionless

The variables introduced in this section constitute the microscopic basis for the Hamiltonian developed in Supplementary Note 2.

S1.4 Microscopic Hamiltonian

Having established the microscopic variables describing the collective polar displacement and vacancy ordering, we now construct the Hamiltonian governing the ferroelectric state. The objective of this section is to derive the effective free-energy functional used in the main manuscript directly from the microscopic degrees of freedom.

The total Hamiltonian is assumed to consist of two physically distinct contributions,

$$H = H_{\mathrm{loc}} + H_{\mathrm{dd}}, \tag{1}$$

where H_{loc} describes the local lattice restoring energy associated with the polar displacement, while H_{dd} represents the long-range dipole–dipole interaction responsible for the cooperative ferroelectric ordering.

Local lattice energy

Within Landau–Devonshire theory, the local potential energy associated with the polar displacement may be expanded as an even polynomial around the high-symmetry reference configuration,

$$H_{\rm loc} = \sum_i \left[\frac{1}{2} a_0 q_i^2 + \frac{1}{4} b q_i^4 + \frac{1}{6} c q_i^6 \right]. \tag{2}$$

Only even powers of q_i appear because the centrosymmetric reference lattice is invariant under inversion ($q_i \to -q_i$). Consequently, odd-order terms are forbidden by symmetry in the absence of an external electric field.

The harmonic coefficient a_0 represents the intrinsic restoring force of the polar mode, while the higher-order coefficients b and c stabilize the energy at finite displacement and allow the emergence of a double-well potential once the quadratic coefficient becomes negative.

Dipole–dipole interaction

Ferroelectricity is a cooperative phenomenon arising from interactions between neighboring dipoles. The corresponding interaction energy is written as

$$H_{\rm dd} = -\frac{1}{2} \sum_{i \neq j} J_{ij} p_i p_j, \tag{3}$$

where J_{ij} denotes the interaction strength between dipoles located at lattice sites i and j. The factor of 1/2 prevents double counting of dipole pairs.

Substituting the microscopic relation

$$p_i = Z^* q_i, \tag{4}$$

gives

$$H_{\rm dd} = -\frac{1}{2} (Z^*)^2 \sum_{i \neq j} J_{ij} q_i q_j. \tag{5}$$

Equation (5) shows that the interaction energy is quadratic in the collective polar displacement. Physically, stronger dipole–dipole coupling reduces the effective restoring force acting on the polar mode and therefore promotes spontaneous polarization.

Reciprocal-space representation

To identify the instability responsible for ferroelectric ordering, the displacement field is transformed into reciprocal space,

$$q_i = \frac{1}{\sqrt{N}} \sum_{\mathbf{k}} q_{\mathbf{k}} e^{i\mathbf{k}\cdot\mathbf{R}_i}, \tag{6}$$

where $\mathbf{R}_i$ denotes the lattice vector of the i-th unit cell.

Substituting Eq. (6) into Eq. (5) yields

$$H_{\rm dd} = -\frac{1}{2} (Z^*)^2 \sum_{\mathbf{k}} J(\mathbf{k}) q_{\mathbf{k}} q_{-\mathbf{k}}, \tag{7}$$

where

$$J(\mathbf{k}) = \sum_j J_{ij} e^{-i\mathbf{k}\cdot(\mathbf{R}_j - \mathbf{R}_i)} \tag{8}$$

is the Fourier transform of the dipole–dipole interaction.

Ferroelectric ordering corresponds to a spatially uniform polarization. Consequently, the instability first develops in the long-wavelength limit ($\mathbf{k} = 0$), for which all local dipoles oscillate coherently with the same phase. Retaining only this dominant contribution gives

$$H_{\rm dd} \approx -\frac{1}{2} (Z^*)^2 J(0) Q^2, \tag{9}$$

where Q is the collective polar coordinate introduced in Supplementary Note S1.1.

Combining the harmonic local energy with the dipole interaction leads to an effective quadratic coefficient

$$A_{\text{FE}} = a_0 - (Z^*)^2 J(0). \tag{10}$$

Equation (10) demonstrates that the competition between the local restoring force and the cooperative dipole–dipole interaction determines the stability of the ferroelectric state. When the interaction term becomes sufficiently strong, the effective stiffness decreases, eventually leading to the softening of the polar mode. In the following section, we show how oxygen-vacancy ordering modifies the interaction strength $J(0)$ and thereby renormalizes the effective polar stiffness.

S1.5 Vacancy-Induced Renormalization of the Polar-Mode Stiffness

The microscopic Hamiltonian derived in the previous section establishes that the stability of the ferroelectric state is governed by the competition between the intrinsic lattice restoring force and the cooperative dipole–dipole interaction. The remaining task is therefore to determine how oxygen-vacancy ordering modifies this interaction during electrical cycling.

The central physical hypothesis of the present work is that oxygen vacancies do not directly generate spontaneous polarization. Instead, vacancy redistribution modifies the local electrostatic environment surrounding the polar oxygen displacement, thereby changing the effective interaction between neighboring dipoles.

This mechanism is illustrated schematically in Fig. 4a of the main manuscript. As electrical cycling progresses, oxygen vacancies gradually evolve from a nearly random distribution toward a more ordered configuration. This redistribution changes the local electric field experienced by the polar mode, leading to a continuous renormalization of the dipole–dipole interaction.

Symmetry Considerations

The vacancy-order parameter introduced in Eq. (S12) satisfies

$$\eta = \frac{n_A - n_B}{n_A + n_B},$$

which changes sign when the two symmetry-related vacancy environments are interchanged. Since the interaction energy must remain invariant under this exchange operation, the effective interaction cannot depend linearly on η. Consequently, the lowest-order symmetry-allowed correction is quadratic in the vacancy-order parameter.

The interaction strength may therefore be expanded as a Taylor series around the disordered state,

$$J(0,\eta) = J_0 + J_2\eta^2 + J_4\eta^4 + \cdots , \tag{11}$$

where

- J_0 represents the interaction strength for a completely disordered vacancy distribution,
- J_2 describes the leading correction arising from vacancy ordering,
- higher-order terms become increasingly small for moderate vacancy ordering.

Retaining only the leading contribution gives

$$J(0,\eta) \approx J_0 + J_2\eta^2. \tag{12}$$

Equation (12) represents the lowest-order phenomenological description consistent with the symmetry of the vacancy-order parameter.

Renormalized Polar Stiffness

Substituting Eq. (12) into the effective harmonic coefficient derived previously,

$$A_{\mathrm{FE}} = a_0 - (Z^*)^2 J(0), \tag{13}$$

gives

$$A_{\mathrm{eff}} = a_0 - (Z^*)^2 \left(J_0 + J_2\eta^2\right). \tag{14}$$

Rearranging the constant and vacancy-dependent contributions separately,

$$A_{\mathrm{eff}} = \left[a_0 - (Z^*)^2 J_0\right] - (Z^*)^2 J_2\eta^2. \tag{15}$$

For convenience, two phenomenological coefficients are introduced,

$$A_0 = a_0 - (Z^*)^2 J_0, \tag{16}$$

and

$$\gamma = (Z^*)^2 J_2. \tag{17}$$

Substituting Eqs. (16) and (17) into Eq. (15) yields

$$A_{\mathrm{eff}} = A_0 - \gamma\eta^2. \tag{18}$$

Equation (18) constitutes the central result of the present theoretical framework.

Physical Interpretation

Equation (18) demonstrates that oxygen vacancies do not act as a direct source of spontaneous polarization. Instead, they continuously reduce the effective restoring force acting on the collective polar mode by strengthening the cooperative dipole interaction.

Three physically distinct regimes emerge naturally.

1. For small vacancy ordering ($\eta \approx 0$),

$$A_{\mathrm{eff}} \approx A_0,$$

   and the polar mode remains relatively stiff.

2. As electrical cycling proceeds, increasing vacancy ordering continuously reduces the effective stiffness,

$$A_{\mathrm{eff}} < A_0,$$

   causing progressive softening of the ferroelectric mode.

3. When the vacancy ordering reaches a critical value, the quadratic stiffness approaches zero,

$$A_{\mathrm{eff}} \to 0,$$

   signaling the onset of the polarization catastrophe discussed in the following section.

The continuous reduction of the effective stiffness predicted by Eq. (18) provides the microscopic origin of the experimentally observed wake-up behavior, including enhanced remanent polarization, increased switching current, reduced switching barrier, and increased dielectric susceptibility.

S1.6 Construction of the Free Energy and Equilibrium Solutions

Having established the vacancy-dependent renormalization of the effective polar stiffness, we now construct the coarse-grained free-energy functional governing the collective polarization state.

The collective coordinate Q introduced in Supplementary Note S1.1 describes the average polar displacement of the crystal. Since the vacancy-induced renormalization modifies only the quadratic stiffness, the free energy can be written directly in terms of the collective coordinate as

$$F(Q,\eta) = \frac{1}{2}(A_0 - \gamma\eta^2)Q^2 + \frac{1}{4}BQ^4 + \frac{1}{6}CQ^6 - EZ^*Q, \tag{19}$$

where

- the quadratic term represents the vacancy-dependent harmonic restoring force,
- the quartic and sixth-order terms stabilize the free energy at finite polar displacement,
- the final term describes the interaction between the external electric field and the collective polarization.

The electric-field contribution follows directly from the electrostatic energy

$$U = -EP,$$

together with the microscopic relation

$$P = \frac{Z^*}{\Omega}Q.$$

After absorbing the constant factor $1/\Omega$ into the normalization of the free-energy density, the field coupling becomes

$$-EZ^*Q.$$

Spontaneous Polarization

The spontaneous ferroelectric state corresponds to the absence of an external electric field. Consequently,

$$E = 0, \tag{20}$$

and Eq. (19) reduces to

$$F(Q,\eta) = \frac{1}{2}(A_0 - \gamma\eta^2)Q^2 + \frac{1}{4}BQ^4 + \frac{1}{6}CQ^6. \tag{21}$$

Equilibrium Condition

The equilibrium polarization corresponds to the stationary point of the free energy,

$$\frac{\partial F}{\partial Q} = 0. \tag{22}$$

Differentiating Eq. (19) gives

$$\frac{\partial F}{\partial Q} = (A_0 - \gamma\eta^2)Q + BQ^3 + CQ^5 - EZ^*. \tag{23}$$

For spontaneous polarization ($E = 0$),

$$(A_0 - \gamma\eta^2)Q + BQ^3 + CQ^5 = 0. \tag{24}$$

Factoring out the common factor Q gives

$$Q\left[(A_0-\gamma\eta^2)+BQ^2+CQ^4\right]=0. \tag{25}$$

Equation (25) immediately yields two classes of solutions.
The first solution,

$$Q=0, \tag{26}$$

corresponds to the nonpolar state.
The second class of solutions satisfies

$$CQ^4+BQ^2+(A_0-\gamma\eta^2)=0. \tag{27}$$

Solution of the Quartic Equation

Introducing the substitution

$$x=Q^2, \tag{28}$$

transforms Eq. (27) into the quadratic equation

$$Cx^2+Bx+(A_0-\gamma\eta^2)=0. \tag{29}$$

Applying the quadratic formula gives

$$x=\frac{-B\pm\sqrt{B^2-4C(A_0-\gamma\eta^2)}}{2C}. \tag{30}$$

Since $x=Q^2$, only non-negative values of x correspond to physically real polarization states. The physically relevant branch is therefore

$$Q_s=\pm\left[\frac{-B+\sqrt{B^2-4C(A_0-\gamma\eta^2)}}{2C}\right]^{1/2}, \tag{31}$$

where the positive and negative solutions represent the two degenerate ferroelectric polarization states.

Using the relation between polarization and the collective coordinate,

$$P=\frac{Z^*}{\Omega}Q, \tag{32}$$

the spontaneous polarization becomes

$$P_s=\pm\frac{Z^*}{\Omega}\left[\frac{-B+\sqrt{B^2-4C(A_0-\gamma\eta^2)}}{2C}\right]^{1/2}. \tag{33}$$

Equation (33) gives the magnitude of the spontaneous polarization. The two solutions $\pm P_s$ correspond to the two symmetry-equivalent ferroelectric states separated by the free-energy barrier.

S1.7 Stability Analysis and Experimental Consequences

The equilibrium solutions derived in Supplementary Note S1.6 correspond to stationary points of the free-energy landscape. However, not every stationary solution is physically stable. The stability of the ferroelectric state is determined by the curvature of the free-energy surface, which is obtained from the second derivative with respect to the collective polar coordinate.

Local Stability Criterion

The second derivative of Eq. (19) is

$$\frac{\partial^2 F}{\partial Q^2} = (A_0 - \gamma\eta^2) + 3BQ^2 + 5CQ^4. \tag{34}$$

A stable equilibrium requires

$$\frac{\partial^2 F}{\partial Q^2} > 0, \tag{35}$$

whereas

$$\frac{\partial^2 F}{\partial Q^2} < 0 \tag{36}$$

corresponds to an unstable stationary point.

Catastrophe Boundary

The onset of the ferroelectric instability occurs when the curvature of the free-energy surface at the origin vanishes.

Evaluating Eq. (34) at

$$Q = 0,$$

gives

$$\left.\frac{\partial^2 F}{\partial Q^2}\right|_{Q=0} = A_0 - \gamma\eta^2 = A_{\text{eff}}. \tag{37}$$

The instability therefore occurs when

$$A_{\text{eff}} = 0. \tag{38}$$

Substituting Eq. (18) gives the critical vacancy-order parameter

$$\eta_c = \sqrt{\frac{A_0}{\gamma}}. \tag{39}$$

Below the critical value,

$$\eta < \eta_c,$$

the free energy contains a single minimum located at

$$Q = 0.$$

For

$$\eta > \eta_c,$$

the quadratic coefficient becomes negative, producing two symmetry-equivalent minima located at

$$Q = \pm Q_s.$$

The continuous evolution from a single-well to a double-well potential constitutes the polarization catastrophe proposed in the present work.

Dimensionless Representation

For convenience, a normalized vacancy-order parameter is introduced,

$$r = \frac{\eta}{\eta_c}. \tag{40}$$

Using Eq. (39),

$$A_{\text{eff}} = A_0(1 - r^2). \tag{41}$$

Equation (41) is employed throughout the numerical calculations presented in Fig. 4 because it removes one fitting parameter while preserving the complete mathematical structure of the model.

Soft-Mode Frequency

Within the harmonic approximation, the squared frequency of the polar mode is

$$\omega_p^2 = \frac{A_{\text{eff}}}{M}, \tag{42}$$

where M denotes the effective mass associated with the collective polar coordinate.
As vacancy ordering increases,

$$A_{\text{eff}} \to 0,$$

and therefore

$$\omega_p \to 0,$$

indicating complete softening of the ferroelectric mode at the catastrophe boundary.

Dielectric Susceptibility

The dielectric susceptibility is proportional to the inverse curvature of the free-energy surface,

$$\chi \propto \frac{1}{A_{\text{eff}}}. \tag{43}$$

Consequently, the susceptibility increases continuously as the catastrophe boundary is approached, leading to the experimentally observed enhancement of the capacitance during wake-up.

Switching Barrier

The energy barrier separating the two polarization states is determined by the difference between the free energy at the unstable saddle point and the stable polar minimum,

$$\Delta U = F(Q = 0) - F(Q_s). \tag{44}$$

As the effective stiffness decreases,

$$A_{\text{eff}} \to 0,$$

the barrier height continuously decreases, facilitating polarization reversal and increasing the switching probability.

Switching Current

The experimentally measured switching current follows directly from the time derivative of the polarization,

$$I_{\rm sw} = A_{\rm dev}\frac{dP}{dt}, \tag{45}$$

where $A_{\rm dev}$ denotes the capacitor area.
Using

$$P = \frac{Z^*}{\Omega}Q,$$

Eq. (45) becomes

$$I_{\rm sw} = A_{\rm dev}\frac{Z^*}{\Omega}\frac{dQ}{dt}. \tag{46}$$

Near the catastrophe boundary, the reduced restoring force allows more rapid polarization reversal, thereby increasing the peak switching current. This prediction is consistent with the experimentally observed increase in switching-current amplitude following wake-up.

Summary

The complete theoretical framework demonstrates that all experimentally observed signatures of wake-up originate from the same microscopic mechanism. Vacancy ordering progressively renormalizes the dipole–dipole interaction, thereby reducing the effective polar stiffness. The resulting softening of the polar mode simultaneously produces the emergence of spontaneous polarization, enhancement of the dielectric susceptibility, collapse of the switching barrier, reduction of the coercive field, and increase in the switching current. The theoretical curves presented in Fig. 4 are generated directly from the equations derived in Supplementary Note S1.

Supplementary Note 2. Numerical Implementation of the Theoretical Model

The analytical expressions derived in Supplementary Note 1 were evaluated numerically to generate the theoretical curves presented in Figs. 4 and 5 of the main manuscript. The calculations were implemented using Python (version 3.12) with the NumPy, SciPy, SymPy, Pandas, and Matplotlib libraries. The complete executable notebook is provided as Supplementary Software [1–4].

The objective of the numerical implementation is not to fit the experimental data directly but to illustrate the physical evolution predicted by the vacancy-assisted polarization-catastrophe model. Unless otherwise stated, all parameters are expressed in normalized units.

S2.1 Parameter Normalization

The effective stiffness derived in Supplementary Note 1 is

$$A_{\rm eff} = A_0 - \gamma\eta^2.$$

Rather than using the vacancy-order parameter directly, a normalized variable

$$r = \frac{\eta}{\eta_c}$$

is introduced, where

$$\eta_c = \sqrt{\frac{A_0}{\gamma}}.$$

Consequently,

$$A_{\text{eff}} = A_0(1 - r^2).$$

This normalization reduces the number of independent fitting parameters and places the polarization catastrophe at

$$r = 1,$$

which provides a convenient reference point for comparing different physical quantities.

S2.2 Choice of Model Parameters

The theoretical curves shown in Fig. 4 were generated using normalized phenomenological parameters,

$$A_0 = 1,$$

$$B = 0.2,$$

$$C = 0.2,$$

$$\gamma = 1.$$

These values were selected to satisfy the stability conditions

$$C > 0,$$

$$B^2 - 4CA_{\text{eff}} > 0,$$

while producing representative free-energy evolution across the catastrophe boundary. The parameters are intended to illustrate the mathematical behavior of the model rather than to represent a quantitative fit to a specific device.

S2.3 Numerical Solution

For each value of the normalized vacancy-order parameter, the equilibrium polarization was obtained by solving

$$CQ^4 + BQ^2 + A_{\text{eff}} = 0.$$

The substitution

$$x = Q^2$$

transforms the quartic equation into a quadratic equation that can be solved analytically. Only solutions satisfying

$$x \geq 0$$

were retained. The spontaneous polarization was subsequently obtained from

$$Q = \pm\sqrt{x}.$$

Imaginary solutions and negative values of x were discarded because they do not correspond to physically realizable polarization states.

S2.4 Computational Workflow

The numerical implementation follows the sequence

1. Define the phenomenological parameters.
2. Calculate the normalized vacancy-order parameter.
3. Evaluate the effective stiffness.
4. Solve the equilibrium equation.
5. Identify the stable polarization branches.
6. Construct the free-energy landscape.
7. Calculate the derived quantities including susceptibility, switching barrier, and soft-mode frequency.
8. Generate publication-quality figures.

The complete executable implementation is supplied as a Google Colab notebook, allowing every theoretical figure in the manuscript to be reproduced without additional software installation.

Table 2: Normalized phenomenological parameters employed in the numerical implementation of the vacancy-assisted polarization-catastrophe model. These parameters were selected to satisfy the stability conditions of the free-energy expansion while illustrating the qualitative evolution of the ferroelectric state.

Parameter	Physical meaning	Value	Unit
A_0	Reference polar-mode stiffness	1.0	Normalized
γ	Vacancy-induced coupling coefficient	1.0	Normalized
B	Quartic Landau coefficient	0.2	Normalized
C	Sixth-order Landau coefficient	0.2	Normalized
η_c	Critical vacancy-order parameter	$\sqrt{A_0/\gamma}$	Dimensionless
r	Normalized vacancy-order parameter ($r = \eta/\eta_c$)	Variable	Dimensionless

Table 3: Computational environment used for the numerical implementation and reproduction of the theoretical figures. The calculations can be executed directly using the accompanying Google Colab notebook without requiring local software installation.

Software / Package	Version	Purpose
Python	3.12 (or later)	Programming language
NumPy	2.x	Numerical array operations
SciPy	1.x	Scientific computing utilities
SymPy	1.x	Symbolic mathematical derivation
Pandas	2.x	Data handling and manipulation
Matplotlib	3.x	Figure generation and plotting
Google Colab	Current release	Cloud-based execution environment

Supplementary Software

The complete Python implementation used to generate all theoretical figures presented in the main manuscript is provided as the accompanying Supplementary Software. The notebook contains the symbolic derivation, numerical implementation, and figure-generation routines described in Supplementary Notes 1 and 2. Executing all cells sequentially reproduces the theoretical results reported in Figs. 4 and 5 without requiring any manual modification of the source code.

Supplementary Note 2. Methods

S2.1. Device fabrication

Metal–ferroelectric–metal (MFM) capacitors consisting of TaN/$Hf_{0.5}Zr_{0.5}O_2$/TiN were fabricated using a complementary metal–oxide–semiconductor (CMOS)-compatible process flow. The bottom TiN electrode was first deposited on a Si substrate, followed by the deposition of a nominally 10 nm $Hf_{0.5}Zr_{0.5}O_2$ (HZO) ferroelectric layer by atomic layer deposition (ALD). A TaN top electrode was subsequently deposited to complete the capacitor stack. Following deposition, the samples were crystallized by rapid thermal annealing (RTA) at 500 °C for 30 s under a nitrogen atmosphere to stabilize the metastable orthorhombic ferroelectric phase. Circular capacitor structures were then defined using standard photolithography and dry-etching processes. The final HZO thickness determined from cross-sectional transmission electron microscopy was approximately 9.5 nm. The TiN bottom electrode served as both the electrical contact and mechanical confinement layer during crystallization, while the TaN top electrode provided the external bias during electrical characterization. All fabrication processes were carried out using semiconductor cleanroom facilities with CMOS-compatible processing conditions.

S2.2. Electrical characterization

Electrical measurements were performed at room temperature using a B1500A Semiconductor Device Parameter Analyzer (Keysight Technologies, USA) equipped with a B1530A Waveform Generator/Fast Measurement Unit (WGFMU). Ferroelectric polarization was characterized using the positive-up-negative-down (PUND) pulse sequence with a standard pulse width of 500 μs. Pulse amplitudes were varied from 1 to 2 V in 0.1 V increments, with additional measurements up to 3 V to investigate the voltage dependence of the switching characteristics. The remanent polarization (P_r) was obtained by integrating the switching current after subtraction of the non-switching current component, while the peak switching current (I_{sw}) and hysteresis-loop area were extracted directly from the measured polarization–voltage characteristics [5, 6].

Wake-up and fatigue behavior were investigated by repeated bipolar electrical cycling. The evolution of remanent polarization, switching current, hysteresis-loop area, and capacitance was monitored after selected switching cycles, while fatigue measurements were performed up to 10^6 polarization reversals under identical electrical conditions.

Capacitance–voltage (C–V) characteristics were measured using the capacitance measurement unit (CMU) integrated within the B1500A at a frequency of 1 kHz using a small-signal AC excitation superimposed on a DC voltage sweep. Both forward and reverse voltage sweeps were recorded to evaluate the evolution of the ferroelectric butterfly hysteresis during wake-up.

Switching kinetics were investigated by varying the PUND pulse width from 1 to 1500 μs while maintaining a constant pulse amplitude. The pulse-width dependence of the remanent polarization was used to evaluate the polarization-switching dynamics and saturation behavior.

S2.3. Structural characterization

The crystallographic evolution of the $Hf_{0.5}Zr_{0.5}O_2$ (HZO) films was investigated using a PANalytical X'Pert Pro MRD X-ray diffractometer (Malvern Panalytical, The Netherlands) equipped with a Cu Kα radiation source (λ = 1.5406 Å). Symmetric θ–2θ diffraction scans were collected at room temperature before and after electrical wake-up cycling. Diffraction peaks were indexed using the reported crystallographic data for the monoclinic ($P2_1/c$), tetragonal ($P4_2/nmc$), and orthorhombic ($Pca2_1$) phases of HZO. Owing to the overlap of the orthorhombic and tetragonal reflections near 30°, XRD was used to monitor the overall structural evolution, while quantitative phase fractions were determined by four-dimensional scanning transmission electron microscopy (4D-STEM). Cross-sectional transmission electron microscopy specimens were prepared using a focused ion beam (FIB) lift-out method. Electron-transparent lamellae with a final thickness of

approximately 70 nm were fabricated by sequential coarse milling followed by low-energy ion polishing to minimize ion-beam-induced damage and preserve the HZO/TiN interfaces.

graphicx

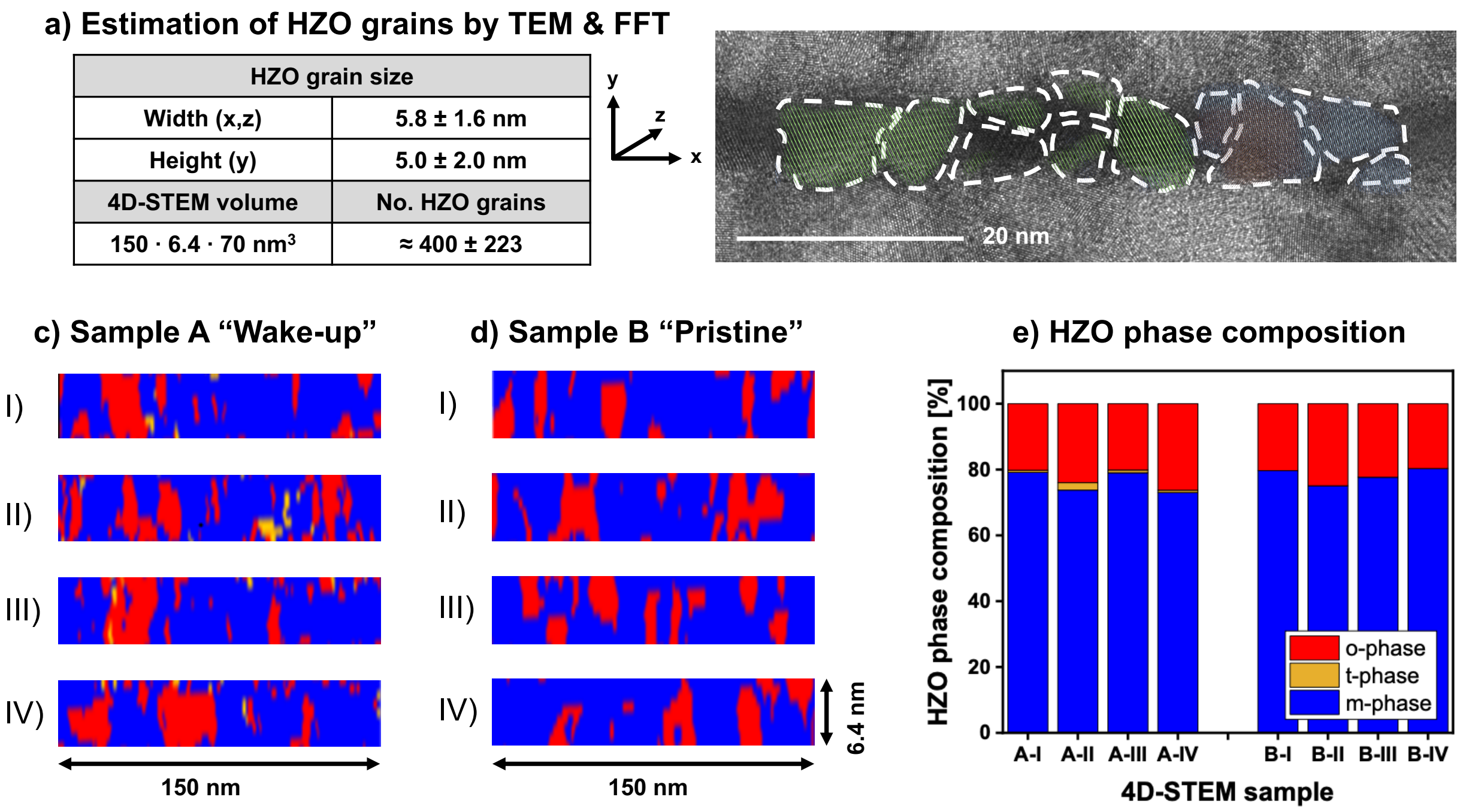

Figure 1: Statistical analysis of HZO thin films by TEM and 4D-STEM. (a) The average HZO grain size is estimated based on identified grain boundaries using TEM imaging and overlaid grain-orientation mapping obtained through FFT analysis. The estimated HZO grain dimensions are used as a guide to determine 4D-STEM map sizes representing a statistically significant number of grains in the investigated TEM sample volume (TEM sample thickness ≈ 70 nm). Four separate 4D-STEM phase maps, each measuring 150 nm × 6.4 nm, are used for the HZO phase-composition analysis of HZO samples after wake-up (b) and in the pristine condition (c). The respective phase compositions of the m-phase (monoclinic, $P2_1/c$), t-phase (tetragonal, $P4_2/nmc$), and o-phase (orthorhombic, $Pca2_1$) are compared for all sampled regions in (e). The corresponding averaged results are presented in Figure 1(a).

Crystal-phase identification was performed using automated crystal orientation mapping (ACOM) based on 4D-STEM diffraction analysis. Experimental diffraction patterns were indexed against simulated templates corresponding to the monoclinic (m, $P2_1/c$), tetragonal (t, $P4_2/nmc$), and orthorhombic (o, $Pca2_1$) polymorphs of HZO. The average HZO grain size was first estimated by combining grain-boundary identification from TEM images with overlaid grain-orientation maps obtained through FFT analysis. These estimated grain dimensions were subsequently used to define 4D-STEM mapping areas containing a statistically significant number of grains within the investigated TEM sample volume, for which the specimen thickness was approximately 70 nm.

Four spatially distinct, non-overlapping 4D-STEM phase maps, each measuring 150 nm × 6.4 nm, were acquired for both the pristine and wake-up states of the TaN/HZO/TiN capacitor structure. The HZO layer thickness was approximately 9.5 nm. Phase fractions were calculated from all successfully indexed pixels within each mapped region and subsequently averaged across the four independently sampled regions. The phase maps shown in Fig. 1(a) of the main manuscript are representative regions selected for visualization, whereas the reported phase fractions correspond to the averaged values obtained from all sampled regions.

The averaged analysis reveals only a modest increase in the orthorhombic phase fraction, from 21.8% ± 2.4% in the pristine state to 22.7% ± 3.0% after wake-up. In parallel, the monoclinic phase fraction decreases from 78.2% ± 2.4% to 76.2% ± 3.0%, accompanied by the emergence of a minor tetragonal component. Representative HRTEM images and diffraction fingerprints used for phase identification are also presented. Although 4D-STEM is intrinsically a spatially localized

characterization technique, the use of multiple independently sampled regions, selected on the basis of the measured grain dimensions, provides a statistically representative assessment of the nanoscale HZO phase composition. The limited variation among the sampled regions, together with the area-averaged XRD response, indicates that the pronounced electrical wake-up is not accompanied by a comparably large crystallographic phase conversion.

The chemical evolution of oxygen-related bonding configurations during wake-up and fatigue was investigated using a ULVAC-PHI Quantes XPS/HAXPES system equipped with monochromatic Al Kα ($h\nu = 1.486$ keV) and Cr Kα ($h\nu = 5.4$ keV) X-ray sources. Before the measurements, the TaN top electrode was completely removed by atomic layer etching. Endpoint detection was used to terminate the etching process at the TaN/HZO interface and minimize overetching or modification of the underlying HZO layer.

High-resolution O 1s spectra were acquired using the monochromatic Cr Kα hard-X-ray source directly from the exposed HZO surface corresponding to the former TaN/HZO interface. Owing to the increased photoelectron kinetic energy, the measured signal contains contributions from a substantial portion of the approximately 9.5 nm HZO layer. However, the photoelectron intensity remains attenuation-weighted and is therefore more sensitive to the upper HZO region adjacent to the former TaN/HZO interface.

The O 1s spectra should consequently be interpreted as depth-integrated, attenuation-weighted chemical-state measurements rather than as depth-resolved oxygen-vacancy profiles. The measurements cannot independently establish whether the cycling-induced oxygen-defect evolution is localized near the former top interface or distributed throughout the complete HZO thickness.

Pristine, wake-up, and fatigued samples underwent identical endpoint-controlled TaN-removal procedures and were measured under identical acquisition conditions. The spectra were charge corrected using the adventitious C 1s reference, followed by Shirley-background subtraction and peak fitting using mixed Gaussian–Lorentzian line shapes with identical fitting constraints. The O 1s spectra were decomposed into lattice-oxygen and oxygen-vacancy-related components. The integrated vacancy-related O 1s spectral fraction was used as a comparative proxy for cycling-induced changes in the oxygen-defect chemical environment within the HAXPES-sensitive HZO volume, rather than as a direct measurement of a spatially uniform bulk oxygen-vacancy concentration.

S2.4. DFT Simulation

To investigate the influence of oxygen vacancies on the ferroelectric polarization of orthorhombic $Hf_{0.5}Zr_{0.5}O_2$ (HZO), ordered vacancy configurations containing one to four oxygen vacancies were constructed, corresponding to different oxygen-deficiency concentrations. Molecular dynamics (MD) simulations were performed using the LAMMPS package [7] accelerated through the KOKKOS performance portability framework [8]. Interatomic interactions were described using the MACE-medium machine-learned interatomic potential [9]. Each structure was equilibrated for 75 ps at 300 K using the NPT ensemble with a Nosé–Hoover thermostat and barostat[10, 11], while periodic boundary conditions were applied along all three crystallographic directions. The MD simulations allowed both the lattice parameters and atomic positions to evolve freely, enabling finite-temperature relaxation and thermally driven redistribution of oxygen vacancies. The MD equilibrated structures were then used as input for first-principles calculations performed within the QuantumATK framework[12]. Density functional theory (DFT) calculations employed the PBEsol exchange-correlation functional together with the FHI Double-Zeta Polarized (DZP) localized basis set. The spontaneous polarization was evaluated using the Berry-phase formalism[13], allowing direct comparison of the polarization before and after finite-temperature equilibration. To facilitate comparison across vacancy concentrations, the polarization magnitude was normalized with respect to the pristine HZO structure.

References

[1] A. Meurer, C. P. Smith *et al.*, "Sympy: symbolic computing in python," *PeerJ Computer Science*, vol. 3, p. e103, 2017.

[2] C. R. Harris *et al.*, "Array programming with numpy," *Nature*, vol. 585, pp. 357–362, 2020.

[3] P. Virtanen *et al.*, "Scipy 1.0: fundamental algorithms for scientific computing in python," *Nature Methods*, vol. 17, pp. 261–272, 2020.

[4] J. D. Hunter, "Matplotlib: A 2d graphics environment," *Computing in Science & Engineering*, vol. 9, pp. 90–95, 2007.

[5] C.-Y. Cho, C.-Y. Chiu, S. De, and T.-H. Hou, "Exploring beol-compatible ferroelectricity in ultra-thin hafnium zirconium oxide: Thermal budget, ftj characteristics, and device reliability," *IEEE Journal of the Electron Devices Society*, 2024.

[6] C.-Y. Chiu, S. De, C.-Y. Cho, and T.-H. Hou, "Trade-off between thermal budget and thickness scaling: A bottleneck on quest for beol compatible ultra-thin ferroelectric films sub-5nm," in *IEEE Electron Devices Technology and Manufacturing Conference (EDTM)*, 2024.

[7] A. P. Thompson, H. M. Aktulga, R. Berger, D. S. Bolintineanu, W. M. Brown, P. S. Crozier, P. J. In't Veld, A. Kohlmeyer, S. G. Moore, T. D. Nguyen *et al.*, "Lammps-a flexible simulation tool for particle-based materials modeling at the atomic, meso, and continuum scales," *Computer physics communications*, vol. 271, p. 108171, 2022.

[8] A. Johansson, E. Weinberg, C. Trott, M. McCarthy, and S. Moore, "Lammps-kokkos: Performance portable molecular dynamics across exascale architectures," in *Proceedings of the SC'25 Workshops of the International Conference for High Performance Computing, Networking, Storage and Analysis*, 2025, pp. 1217–1232.

[9] I. Batatia, P. Benner, Y. Chiang, A. M. Elena, D. P. Kovács, J. Riebesell, X. R. Advincula, M. Asta, M. Avaylon, W. J. Baldwin *et al.*, "A foundation model for atomistic materials chemistry," *The Journal of chemical physics*, vol. 163, no. 18, 2025.

[10] S. Nosé, "A molecular dynamics method for simulations in the canonical ensemble," *Molecular physics*, vol. 52, no. 2, pp. 255–268, 1984.

[11] W. G. Hoover, "Canonical dynamics: Equilibrium phase-space distributions," *Physical review A*, vol. 31, no. 3, p. 1695, 1985.

[12] S. Smidstrup, T. Markussen, P. Vancraeyveld, J. Wellendorff, J. Schneider, T. Gunst, B. Verstichel, D. Stradi, P. A. Khomyakov, U. G. Vej-Hansen *et al.*, "Quantumatk: an integrated platform of electronic and atomic-scale modelling tools," *Journal of Physics: Condensed Matter*, vol. 32, no. 1, p. 015901, 2020.

[13] R. King-Smith and D. Vanderbilt, "Theory of polarization of crystalline solids," *Physical Review B*, vol. 47, no. 3, p. 1651, 1993.
